# Effects of Nucleoside Analogue on Patients with Chronic Hepatitis B-Associated Liver Failure: Meta-Analysis

**DOI:** 10.1371/journal.pone.0054773

**Published:** 2013-01-28

**Authors:** Feng Xie, Long Yan, Jiongjiong Lu, Tao Zheng, Changying Shi, Jun Ying, Rongxi Shen, Jiamei Yang

**Affiliations:** Department of Special Treatment and Liver Transplantation, Eastern Hepatobiliary Surgery Hospital, The Second Military Medical University, Shanghai, China; Yonsei University College of Medicine, Republic Of Korea

## Abstract

**Purpose:**

The effectiveness of nucleoside analogue on patients with chronic hepatitis B-associated liver failure is still controversial. To address this issue, we did a review of the literatures and analyzed the data with emphasis on the survival and reduction in serum HBV DNA level.

**Methods:**

We searched 11 randomized controlled trials that included 654 patients with chronic hepatitis B-associated liver failure. 340 patients adopted nucleoside analogue, such as lamivudine (LAM), entecavir (ETV), telbivudine (LdT), or tenofovir disoproxil fumarate (TDF), and the remaining 314 patients adopted no nucleoside analogue or placebo. A meta-analysis was carried out to examine the survival, HBV e antigen serologic conversion, and reduction in serum HBV DNA level. The pooled odds ratio (OR) was used to reflect the treatment effects.

**Results:**

The overall analysis revealed nucleoside analogue significantly improved 1-month(OR = 2.10; 95% CI, [1.29, 3.41]; p = 0.003), 3-month (OR = 2.15; 95% CI, [1.26, 3.65]; p = 0.005), 12-month survival (OR = 4.62; 95% CI, [1.96, 10.89]; p = 0.0005). Comparison of 3-month HBV DNA showed significant reduction for adoptive nucleoside analogue patients (OR = 54.47; 95% CI, [16.37, 201.74]; p<0.00001). Comparison of 3-month HBV e antigen serologic conversion showed a highly significant improvement of HBV e antigen lost for patients received adoptive antiviral therapy (OR = 6.57; 95% CI, [1.64, 26.31]; p = 0.008).

**Conclusions:**

The benefits of nucleoside analogue on patients with chronic hepatitis B-associated liver failure is significant for improving patient survival, HBV e antigen serologic conversion, and rapid reduction of HBV DNA levels.

## Introduction

Chronic hepatitis B(CHB),caused by the hepatitis B virus (HBV), is a serious health problem worldwide, especially in China and other parts of Asia[1]. Chronic HBV infection is the most common cause of liver failure, which can develop as acute liver failure (ALF) (in the absence of any pre-existing liver disease), acute-on-chronic liver failure (ACLF) (an acute deterioration of known or unknown chronic liver disease), or a chronic decompensation of an end-stage liver disease[2]. Liver failure is a clinical syndrome that the major liver functions, particularly detoxification, synthetic functions and metabolic regulation, are impaired and it can lead to hepatic encephalopathy, ascites, jaundice, cholestasis, bleeding and hepatorenal syndrome (HRS)[3]. If the patients could not received effective treatments, they get poor prognosis because of the severity of the disease and the presence of active viral replication, which is considered as a determinant of prognosis recently[4].

As oral antiviral agents, nucleoside analogue, including lamivudine (LAM), adefovir dipivoxil (ADV), entecavir (ETV), telbivudine (LdT), and tenofovir disoproxil fumarate (TDF), have activities conferring biochemical, virological, and serological improvement in CHB patients[5], [6], and then generate the function of preventing cirrhosis and, consequently, liver failure and hepatocellular carcinoma, even for the decompensated liver disease patients[7].

Recently nucleoside analogue have been proved efficacious in improving the status of patients with severe decompensated chronic liver disease and acute-on-chronic liver failure (ACLF) related with CHB[8]–[13]. However, some effects for these patients are less conclusive, like the effect generated by using nucleoside analogue for a short-term time. Some studies reported they did not produce significant biochemical changes and slow down the progression of liver failure on patients with severe CHB liver disease and acute exacerbation of CHB[14]–[16]. Hence, we selected some randomized controlled trials (RCTs), and then conducted a review and meta-analysis to evaluate the efficacy of nucleoside analogue treatment on chronic liver failure patients.

## Methods

### Selection criteria

In the meta-analysis, we included studies from randomized controlled trials that compared the antiviral therapy adopted nucleoside analogue (included LAM, ADV, ETV, LdT, TDF) with no antiviral treatment for patients who were undergoing chronic hepatitis B-associated liver failure.

Though the concept of acute-on-chronic liver failure (ACLF) is well identified and the diagnostic criteria are unified[2], there isn’t a clear general definition of liver failure induced by chronic hepatitis B. According to the consensus recommendations of the Asian Pacific Association for the study of the liver (APASL) about the acute-on-chronic liver failure, the criteria of chronic hepatitis B-associated liver failure and severe chronic hepatitis B formulated by Chinese Medical Association[17], [18], we made a criteria for the patients with chronic hepatitis B-associated liver failure in the selected studies. Studies were included when the patients met the following criteria:

1. previously diagnosed CHB or HBV induced cirrhosis;
2. progressively raising in serum bilirubin level (>85 µmol/L or >5 mg/dL);
3. prothrombin activity<40% or international normalized ratio (INR)≥1.5;
4. HBV DNA level >10^3^ copies/ml;

And the exclusion criteria were:

1. superinfection with human immunodeficiency virus(HIV) and/or other hepatitis viruses (hepatitis E, A, D, or C);
2. other causes of chronic liver failure, like drug hepatitis, autoimmune hepatitis, alcoholic liver disease, et al;
3. patients were receiving antiviral therapy at the moment when they were recruited or had received antiviral therapy in 6 months before the studies;
4. evidence of hepatocellular carcinoma (HCC);
5. patients had received artificial liver treatment during the studies

### Search strategy

The investigators wrote a protocol and carried out a comprehensive search of Medline, PubMed, Embase, the Cochrane Center Register of Controlled Trials, Biological Abstracts, China National Knowledge Infrastructure, and the Chinese BioMedical Literature Database without language, publication, or date restrictions. However, when the Chinese literatures have no English abstracts, they would be excluded. In addition, reference lists of the trials selected before and relevant reviews were examined for other eligible trials. We also searched http://www.ClinicalTrials.gov website for the information of prospective and ongoing trials. Through the searching task, we used the terms ‘nucleoside analog’,’ nucleoside analogue’,’ nucleotide analog’,’ nucleotide analogue’,’ lamivudine’,’ adefovir dipivoxil’,’ entecavir’,’ telbivudine’,’ tenofovir disoproxil fumarate’,’ liver failure’,’ hepatic failure’,’ chronic hepatitis B’, and ‘severe chronic hepatitis B’.

### Data extraction and quality assessment

Data were extracted independently by two authors, Feng Xie and Long Yan. We screened abstracts of all retrieved articles and then matched the full texts of all articles selected during screening against the inclusion criteria. Disagreements on which articles met the inclusion criteria were resolved by discussion until a consensus was reached. Feng Xie and Long Yan completed the data extraction using a standardized approach that gathered the publication details and study characteristics: year of publication, the first author, number of patients, sex, methods and design, serum HBV DNA level and HBV DNA negative patients, seropositivity and seronegativity for HBV e antigen (HBeAg), alanine transarninase, albumin, serum bilirubin, prothrombin activity or international normalized ratio (INR), number of patients assessable for 1-, 3- and 12-month overall survival.

We used the Jadad score (maximum number of points is 5)[19] to assess the quality of the selected studies based on the description of adequate sequence generation, double blinding, description of deviations and withdrawals. The score was 4 for one study, 3 for one study and 2 for nine studies

### Statistical analysis

The analyses were carried out using STATA 11.0 and Review Manager 5. P values at<0.05 were regarded as statistically significant. Since the study carried out by Yao-Li Cui had a three-arm design: it separately compared two regimens (ETV and LAM) to placebo, each antiviral arm was paired to the control arm independently[32].

The data of 1-, 3- and 12-months survival, patients with negative HBV DNA level, HBV e antigen serologic conversion in each arm were extracted from each study and combined using analysis method named Mantel and Haenszel [20] to calculate the pooled odds ratio (OR). The odds ratios (ORs) reflected the treatment effects. A pooled OR>1 indicated higher survival, HBV DNA negative and HBV e antigen serologic conversion in the antiviral arm. In order to identify the differences of effect of each nucleoside analogue, the subgroup analyses were adopted when the analysis was carried out.

Cochran’s Q test(Chi-square X^2^ test) and I^2^ test were used for across studies to assess the variation across study results that is due to heterogeneity rather than chance. I^2^ can be readily calculated from a typical meta-analysis as I^2^ = 100%×(Q−df)/Q, where Q is Cochran’s heterogeneity statistic and df is the degrees of freedom. Negative values of I^2^ are equal to zero. A value of 0% indicates no observed heterogeneity, and larger values show increasing heterogeneity[21]. In our meta-analysis, P>0.01 in Cochran’s Q test and I^2^<25% would be considered as there was no or low-level heterogeneity, and the fixed-effects model was adopted when the data across studies was pooled. Otherwise, the random-effects model was used. I^2^>50% was considered as significant heterogeneity. In addition to the use of subgroup and the random-effects model, some explanation would be made. Publication bias was assessed visually using a funnel plot.

## Results

### Selection of studies

**Figure 1:**
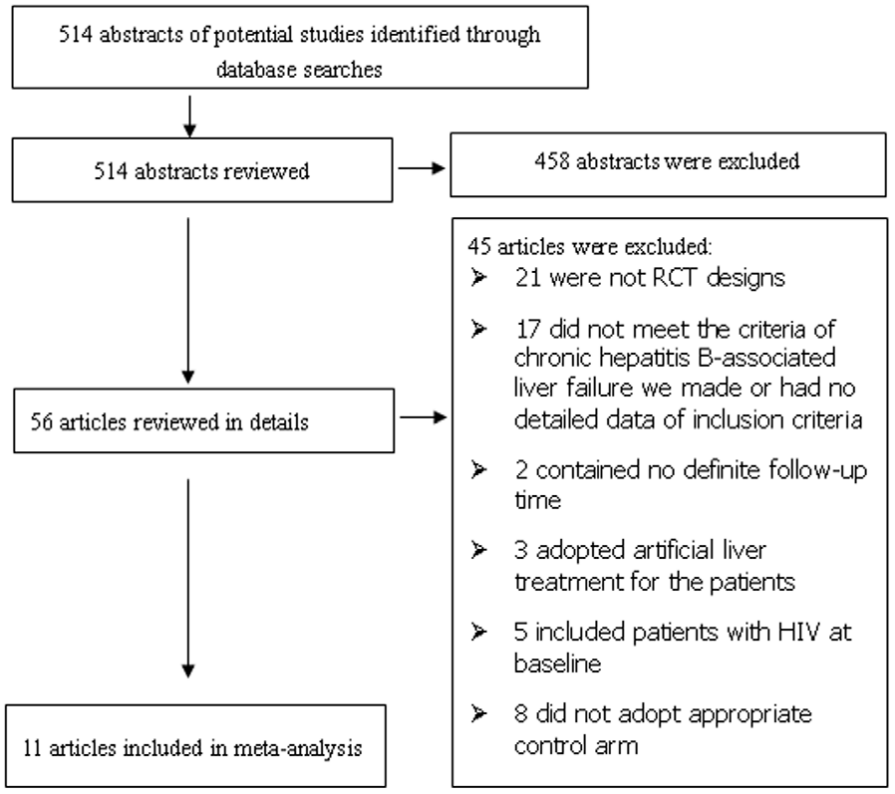
Identification process for eligible studies .

A total of eleven studies met the inclusion criteria for this review[22]–[32], including 654 patients. The strategy summarized in Figure 1. Ten of these studies were from mainland China[22]–[30], [32], and the remaining one[31] was from India. The patients of control arm in ten studies were required to take no nucleoside analogue, and only one[31] adopted placebo as the treatment of control arm. The oral antiviral agents adopted for the patients of the test groups were: 6 studies used ETV[22]–[26], [32], 3 used LAM[27], [28], [32], 2 used LdT[29], [30], and 1 used TDF[31]. One study[32] was three-arm design, and it had two test groups: one used LAM, another used ETV. In addition, excepted liver transplantation and artificial liver treatment, all patients were given standard medical treatment: intensive and care monitoring; supplements of enteral or parenteral nutrition; intravenous drop infusion albumin and plasma; maintenance water, electrolyte and acid–base equilibrium; prevention and treatment complications; etc. The characteristics of each study are listed in Table 1.

**Table 1:** Characteristics of included studies.

Study		Country	Study design	Blinding	Treatment	Number of patients	Age (mean±SD)	Sex (M:F)	HBeAg (positive)	PTA (mean±SD) (%)	INR
**1**	Zhong et al.(2009)	China	RCT	NA	entecavir (ETV)0.5 mg/d	30	NA	20∶10	NA	31.41 ± 5.08	NA
					no nucleoside analogue	30	NA	20∶10	NA	30.94 ± 5.87	NA
**2**	Zhao et al.(2009)	China	RCT	NA	entecavir (ETV)0.5 mg/	40	48.6	54∶26	NA	<40	NA
					no nucleoside analogue	40	48.6	54∶26	NA	<40	NA
**3**	Yang et al.(2008)	China	RCT	NA	entecavir (ETV)0.5 mg	55	28.5±8.9	72∶38	NA	28.5 ± 13.8	NA
					no nucleoside analogue	55	28.5±8.9	72∶38	NA	29.2 ± 12.8	NA
**4**	Guo et al.(2008)	China	RCT	NA	entecavir (ETV)0.5 mg/	37	34.8±8.5	27∶10	18	31.3±6.1	NA
					no nucleoside analogue	45	36.4±9.1	29∶16	20	30.1±7.4	NA
**5**	Han et al.(2009)	China	RCT	NA	entecavir (ETV)0.5 mg/	20	NA	30∶6	NA	28.8±9.5	NA
					no nucleoside analogue	16	NA	30∶6	NA	29.6±9.7	NA
**6**	Xu et al.(2004)	China	RCT	NA	lamivudine (LAM)100 mg/d	11	46.2±7.8	16∶7	NA	38.31±5.69	NA
					no nucleoside analogue	12	46.2±7.8	16∶7	NA	35.66±2.98	NA
**7**	Guo et al.(2003)	China	RCT	NA	lamivudine (LAM)100 mg/d	24	38±17.1	24∶0	24	29.3±8.3	NA
					no nucleoside analogue	24	39±18.4	24∶0	24	29.1±8.4	NA
**8**	Qiu et al.(2009)	China	RCT	NA	telbivudine (LdT)600 mg/d	30	41.45±10.9	48∶12	21	34.29±22.29	NA
					no nucleoside analogue	30	41.45±10.9	48∶12	20	35.58±24.23	NA
**9**	Qin et al.(2012)	China	RCT	NA	telbivudine (LdT)600 mg/d	12	48.65±7.3	15∶9	NA	31.2±9.4	NA
					no nucleoside analogue	12	48.65±7.3	15∶9	NA	29.4±7.6	NA
**10**	Hitendra Garg et al.(2011)	India	RCT	Double Blinding	tenofovir disoproxil fumarate (TDF)300 mg/d	14	47.5	10∶4	8	NA	1.85
					placebo	13	45	10∶3	7	NA	1.93
**11**	Yao et al.(2010)	China	RCT	NA	entecavir (ETV)0.5 mg/d	33	38.39±10.82	3∶30	10	NA	2.27±0.55
					lamivudine (LAM)100 mg/d	34	39.35±10.61	3∶31	13	NA	2.61±1.03
					no nucleoside analogue	37	41.03±11.48	6∶31	11	NA	2.33±0.88

### Survival

**Figure 2:**
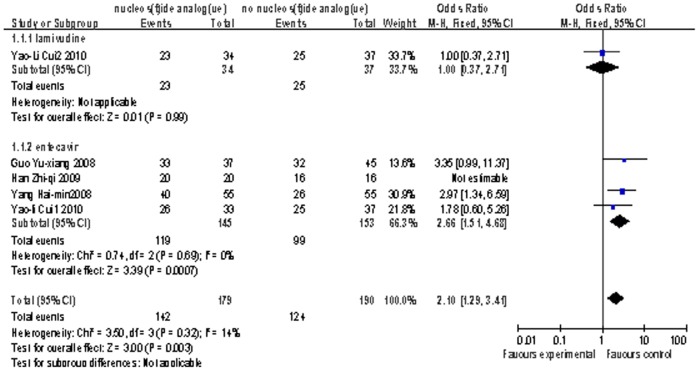
Comparison of 1-month survival of liver failure patients treated with nucleoside analogue or no nucleoside analogue .

Four studies[24]–[26], [32] reported the information of 1-month survival contained 332 patients (179 patients took nucleoside analogue). 145 patients in four studies used ETV and 34 patients used LAM. There was no person died in one study during the first 1 month[26]. The only test arm using LAM presented the same survival rate compared to control arm. The estimated pooled OR for both four studies showed a highly significant survival rate for patients receiving adoptive antiviral therapy (OR = 2.10; 95% CI, [1.29, 3.41]; p = 0.003; Figure 2). To assess the heterogeneity, the Cochran’s Q test showed P = 0.32 and I^2^ test had a value of 14%, what indicated the degree of variability between studies was consistent with what could be estimated to occur by chance. In order to identify the difference of treatment effectiveness between LAM and ETV, the subgroup analysis was adopted. The study in LAM subgroup showed no different survival rate between the test and control arms. The ETV subgroup presented even more highly significant survival rate for patients receiving antiviral therapy (OR = 2.66; 95% CI, [1.51, 4.68]; p = 0.0007), and the P = 0.69 in Cochran’s Q test and I^2^  = 0% when evaluated the heterogeneity.

**Figure 3:**
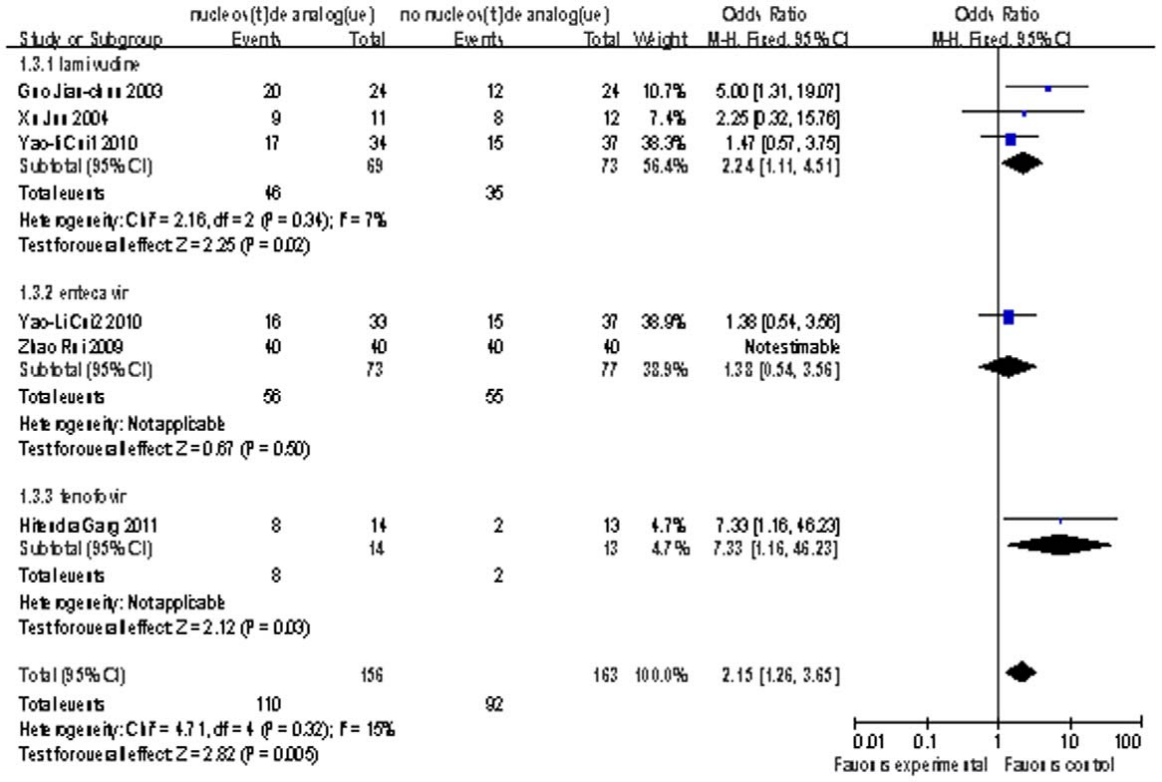
Comparison of 3-month survival of liver failure patients treated with nucleoside analogue or no nucleoside analogue .

Five studies[23], [27], [28], [31], [32] reported the information of 3-month survival including 282 patients (156 patients took nucleoside analogue). Among these studies, LAM was used in three studies (69 patients), ETV was used in two studies (73 patients), and TDF was used in one study (14 patients). The study carried out by Zhao Rui had no person died in the first 3 months. The estimated pooled OR for five studies showed a highly significant survival rate for patients receiving antiviral therapy (OR = 2.15; 95% CI, [1.26, 3.65]; p = 0.005; Figure 3). The assessment of heterogeneity acquired P = 0.32 in Cochran’s Q test and I^2^ = 15%, meaning there was no significant variability of these studies. The subgroup of ETV and TDF had individual OR value of 1.38 (95% CI, [0.54, 3.56]) and 7.33 (95% CI, [1.16, 46.23]). The OR value was 2.24 (95% CI, [1.11, 4.51]) in the LAM subgroup, and it had no evidence of significant heterogeneity (P = 0.34 in Cochran’s Q test and I^2^ = 7%).

**Figure 4:**
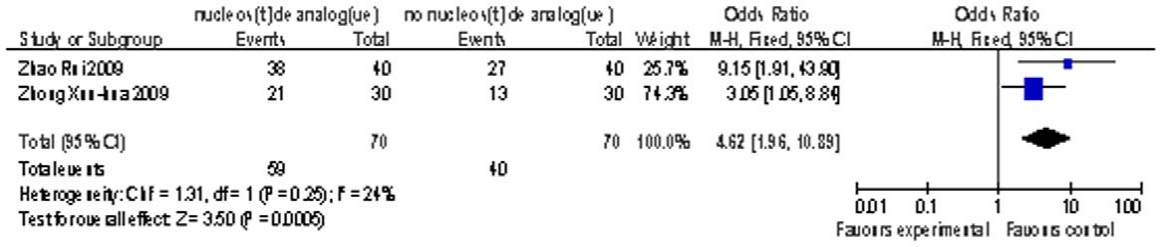
Comparison of 12-month survival of liver failure patients treated with nucleoside analogue or no nucleoside analogue .

There were only two studies[22], [23] reported the information of 12-month survival contained 140 patients. All the two studies adopted ETV as the oral antiviral agents for 70 patients. The result also indicated higher survival rate compared nucleoside analogue arms with control arms (OR = 4.62; 95% CI, [1.96, 10.89]; p = 0.0005; Figure 4).It had no evidence of significant heterogeneity (P = 0.25 in Cochran’s Q test and I^2^  = 24%).

### Reduction in serum HBV DNA level

**Figure 5:**
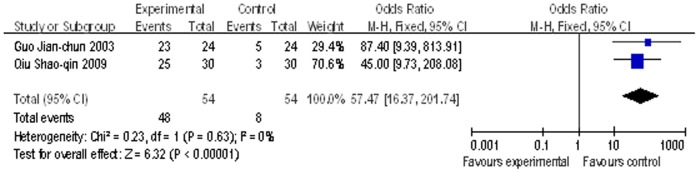
Comparison of 3-month HBV DNA reduction of liver failure patients treated with nucleoside analogue or no nucleoside analogue .

Four studies[27]–[29], [31] reported the information of 3-month HBV DNA reduction. The concept of negative HBV DNA was used to assess the effects of reduction in serum HBV DNA, which meant the patients with undetectable HBV DNA level follow the lowest detection limit of the test equipment. The lowest detection limit was not mentioned in one study[27]. There was one study[31] used the lowest detection limit of 50 IU/mL (about 280 copies/mL), and at 3 months, undetectable HBV DNA was achieved in three of eight (37%) patients in the TDF-treated group (eight persons were survival at the end of 3 months), whereas none in the placebo group. The remaining two studies used the lower detection limit of 10^3^ copies/mL (about 178 IU/mL). The pooled OR for these two studies of negative HBV DNA patients showed significant reduction in HBV DNA for adoptive nucleoside analogue patients (OR = 54.47; 95% CI, [16.37, 201.74]; p<0.00001; Figure 5). There was no evidence of heterogeneity among the two individual studies (p = 0.63 in Cochran’s Q test; I^2^ = 0%).

### HBV e antigen serologic conversion

**Figure 6:**
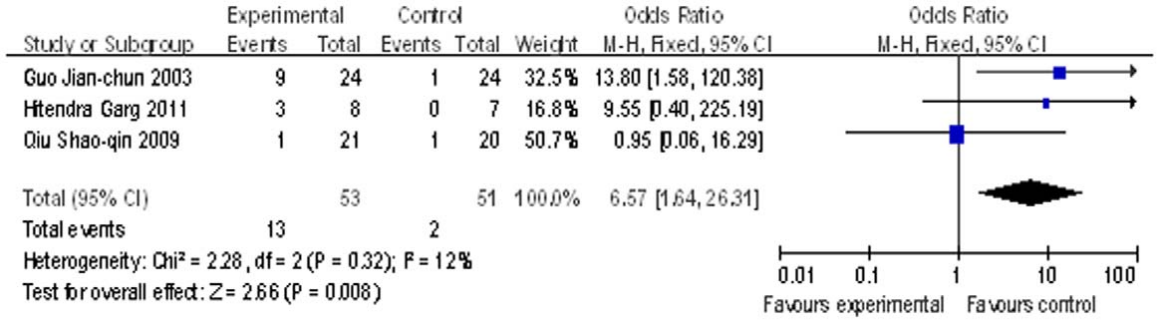
Comparison of 3-month HBV e antigen serologic conversion of liver failure patients treated with nucleoside analogue or no nucleoside analogue .

Three studies [28], [29], [31] reported the information of 3-month HBV e antigen serologic conversion. There was one study used LAM as the oral antiviral agents (including 24 patients) and one study used LdT (including 30 patients). The remaining one having 14 patients used TDF. According to patients lost HBV e antigen in these three studies, the estimated pooled OR value showed a highly significant improvement of HBV e antigen lost for patients received adoptive antiviral therapy (OR = 6.57; 95% CI, [1.64, 26.31]; p = 0.008; Figure 6). It had no evidence of significant heterogeneity in these three studies (P = 0.32 in Cochran’s Q test and I^2^ = 12%).

### Safety

None of these studies reported patients developed significant adverse reaction, or need for dose modification, early discontinuation. Only one study observed two patients had mild gastrointestinal reaction after they took ETV[25], and that was tolerated well and did not disturb the therapy.

### Publication bias

**Figure 7:**
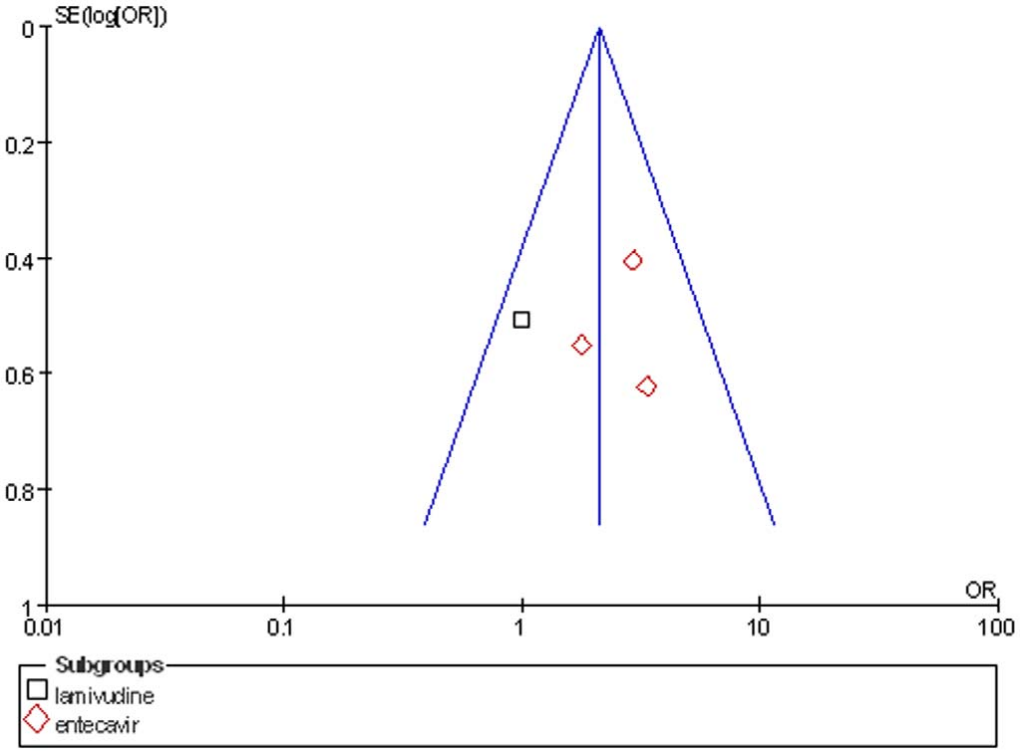
The funnel plot of OR value for 1-month survival of liver failure patients .

A funnel plot of the studies used in the meta-analysis reporting on 1-month survival is shown in Figure 7. None of the studies lay outside the limits of the 95% CI, and there was no evidence of publication bias.

## Discussion

The result clearly indicated nucleoside analogue significant improve survival, HBV e antigen serologic conversion, and rapid reduction of HBV DNA levels. The effects of nucleoside analogue for severe liver disease have been demonstrated by plenty of previous trails. But most of them were not RCTs, and the conclusions of many articles, even guidelines, originated from them. What we wanted to do was to get a stricter conclusion. Now that liver failure is considered as a complication of chronic hepatitis B infection, and of course it would provoke concern in recruiting a sufficient number of patients to conduct a controlled trial using no antiviral treatment (even in areas of high endemicity). What’s more, for patients with chronic hepatitis B-associated liver failure, liver transplantation is currently regarded as a primary and complementary measure[33], and withholding antiviral treatment would have been considered as unethical, which also made the RCTs have not enough participants. However, in China and India, liver transplantation is not readily available nor feasible due to the lack of organs and facilities for liver transplantation. This had made some RCT studies were conducted in these areas.

For patients with severe liver disease associated with CHB, the severity of liver disease at the time of initiating antiviral therapy, like elevated base-line serum bilirubin and creatinine levels and detectable baseline serum HBV DNA level, is a more relevant determinant of short-term mortality than the virological response[34] That may be the reason for not all patients got benefits from nucleoside analogue treatment.

In our selected RCTs, eight of the eleven searched studies published the survival data after using nucleoside analogue for a short-term time. All of the eight studies showed positive results. Nucleoside analogue can markedly suppress HBV replication by suppression of HBV-polymerase activity, leading to improvement of liver function and reduced incidence of fibrosis, cirrhosis in CHB patients[7], [35]. Recent data suggest that the prognosis of patients with chronic hepatitis B-associated severe liver disease may be related to pretreatment HBV DNA load[36]. The rapidly suppression of HBV DNA load can stabilize or halt disease progression, and thereby improves prognosis, despite when the heightened immune response in the liver is ongoing[32]. From our analysis we found nucleoside analogue also had significant positive effects for the short-term HBV DNA reduction. Probably it caused the improvement of liver function and even the benefit of survival.

Data regarding the efficacy of long-term treatment with nucleoside analogue for those patients with liver failure was lack in previous literatures. The severity of liver disease, the poor prognosis of patients and the high mortality rate[37] has restrict the conducted of long-term trails. One previous study followed up patients who suffered from severe acute hepatitis B for at least one year to observe the effect of LAM, found that there was no significant difference in the clinical outcome, biochemical and HBV e antigen serologic conversion between the LAM treated and the placebo treated groups[13]. Two of our selected studies had the data of 12-month survival, and when compared with no antiviral treatment group, the nucleoside analogue treated group had a higher survival rate (OR = 4.62). However, these two studies were insufficient. Definite conclusion should be further studied and discussed. Especially the risks of drug resistance on these patients could only be acquired from long-term studies.

When we compared the effects of nucleoside analogues, the strategy of subgroup was adopted in our statistical analysis. But the selected studies can only provide the data of two or three nucleoside analogues during the same period of time, the comparision of effects brought by each drugs could not obtain a definite conclusion. Previously some nonRCT studies had provided information about the effects of nucleoside analogue drugs for patients with HBV-related decompensated liver disease[8], [12], [13], [16], [38]. Though all of these nucleoside analogue drugs have been confirmed having positive effects of improvement on survival rate, liver functions, reduction of HBV DNA, for the severe liver disease patients, When taking efficacy and drug resistance into consideration, ETV and TDF would be chosen for the first-line therapy[38]. Due to the weaker activity against LAM-resistant hepatitis B virus of ETV, and the fact that patients with LAM resistance would get a high rate of drug resistance if using ETV 1 mg/day for long-term, TDF could be considered as a better choice than ETV for LAM experienced patients.

When initiating antiviral therapy, the severity of liver disease, like elevated base-line serum bilirubin and creatinine levels and detectable baseline serum HBV DNA level could affect the results of patients[14], [16], [34]. It suggested us that antiviral therapy should be initiated as early as possible before the liver disease become too severe to be rescued.

In conclusion, though liver transplantation is currently regarded as a primary measure for patients with chronic hepatitis B-associated liver failure, the early initiation of adaptive nucleoside analogue drugs for antiviral therapy is also necessary.

Finally, we had to mention the weaknesses of these selected studies. The low quality and the conducted areas must be the main shortages. When they were assessed using the Jadad score, the score was 4 for one study, 3 for one study and 2 for nine studies. And all the studies belonged to Asia (ten studies belonged to the mainland of China, and one belonged to India). Obviously these weaknesses could bring some limitations. Under ideal conditions, more double blinding, and large scale randomised control trials should be carried out.
